# Perceptual discrimination of complex objects: Apolipoprotein E e4 gene‐dose effects in mid‐life

**DOI:** 10.1002/alz.70246

**Published:** 2025-06-10

**Authors:** Claire Lancaster, Sam Berens, Jessica Daly, Jennifer Rusted, Chris M. Bird

**Affiliations:** ^1^ School of Psychology University of Sussex Brighton UK; ^2^ Current address: Brighton & Sussex Medical School University of Sussex Brighton UK

**Keywords:** ageing, APOE, cognition, genetic risk, perceptual discrimination

## Abstract

**INTRODUCTION:**

Complex perceptual discrimination is supported by tau‐vulnerable regions of the medial temporal lobe (MTL); notably the perirhinal cortex. This research tests whether there is a gene‐dose effect of apolipoprotein E (APOE) e4 on perceptual discrimination in mid‐life.

**METHODS:**

Three hundred and thirteen adults (45–65 years; grouped by APOE e4 gene‐dose (142 APOE33, 135 APOE34, 36 APOE44)) completed a Greebles “odd‐one‐out” task.

**RESULTS:**

APOE44 carriers were significantly less accurate in their perceptual judgments than APOE33 and APOE34 individuals. There was a significant Age x APOE e4 gene‐dose interaction in response speed, with the slope of age‐related slowing increasing stepwise with the number of e4 alleles carried. Estimates suggest high‐risk individuals are quicker than the APOE33 control group until age 54, but slower thereafter.

**DISCUSSION:**

Perceptual disadvantages specific to APOE44 individuals suggest this high‐risk group show compromised MTL function by mid‐life, potentially through accelerated tau‐aggregation. Task performance in APOE34 carriers is relatively preserved in mid‐life.

**Highlights:**

- APOE44 carriers show impaired perceptual discrimination in mid‐age.
- APOE34 carriers show preserved perceptual discrimination in mid‐age.
- Age‐related slowing of perceptual judgments increases with APOE e4 gene‐dose.
- Perceptual discrimination tasks may be a valuable early marker of AD risk.

## 1 BACKGROUND

To progress interventions targeting the early, clinically “silent” stages of Alzheimer's disease (AD), the field needs sensitive and scalable tools for identifying individuals at risk of future neurodegeneration. Established neuropsychological tests are critical for diagnosing and monitoring the progression of dementia. However, cognitive markers of preclinical AD may be best identified by considering the topographical staging of AD neuropathology and identifying which cognitive functions are supported by brain regions with early vulnerability. Such markers must be capable of detecting subtle differences in outwardly healthy individuals, as well as predicting further AD‐biomarker progression, and later conversion to dementia.^1^, ^2^, ^3^ Complementing standardized neuropsychological assessment with more targeted, often digitalized, cognitive assessment may enhance our ability to capture the earliest signs of neurodegeneration and refine intervention strategies.^4^, ^5^, ^6^

Tests of perceptual discrimination, specifically those requiring participants to distinguish between representations with a large proportion of overlapping visual features, are a candidate cognitive marker of AD risk. Such measures are sensitive to disadvantages in individuals with mild cognitive impairment and AD.^7^, ^8^ Indeed, older adults without a dementia diagnosis who have been classified as “high‐risk” based on their neuropsychological test scores show impaired ability to distinguish between “Greebles” – abstract 3D‐presented objects.^9^, ^10^ Perceptual discrimination is supported by regions of the medial temporal lobe (MTL), including the anterolateral entorhinal cortex (alERc) and perirhinal cortex (PRc),^11^, ^12^, ^13^, ^14^ with the PRc believed to act as an interface between the ventral visual processing stream, higher‐level perceptual, and memory systems.^15^, ^16^ Given neurofibrillary tau pathology initiates in the PRc, before spreading into the entorhinal and hippocampal regions of the MTL,^17^, ^18^ perceptual discrimination tasks may be a valuable indicator of “preclinical” AD. However, there is no evidence to date establishing the utility of such measures for detecting differences in mid‐age adults with early tau pathology.

The apolipoprotein (APOE) e4 variant is the leading genetic risk factor for sporadic AD,^19^ yet there remains considerable debate as to when in the lifespan detrimental genotype effects emerge and via which mechanisms.^4^, ^20^, ^21^, ^22^ Consistent with APOE e4 promoting earlier toxic gain of neurofibrillary tau,^23^, ^24^, ^25^ complex perceptual discrimination tasks are expected to be sensitive to genotype disadvantages by mid‐life. However, to date there has been limited investigation as to whether APOE e4 carriers show impaired perceptual discrimination, with equivalent performance reported between APOE e4 carriers and an APOE33 control group in early adulthood, including across multiple stimulus modalities.^26^ Middle‐aged individuals with a family history of dementia, however, show disadvantages on a “Greebles” odd‐one‐out task,^27^ supporting a genetic link.

**RESEARCH IN CONTEXT**

1. **Systematic review**: A review of the literature (e.g., PubMed; Google Scholar databases) revealed perceptual discrimination is impaired in the mild to moderate stages of Alzheimer's disease. No prior study, however, tests for an effect of apolipoprotein E (APOE) e4 gene‐dose in healthy adulthood.
2. **Interpretation**: Homozygous carriers of an APOE e4 genetic risk variant show perceptual disadvantages in mid‐life. This may possibly reflect compromised medial temporal lobe (MTL) function in this group, consistent with the theory perceptual discrimination will be vulnerable to early transentorhinal tau‐accumulation. There is limited evidence of impairment in individuals carrying one copy of APOE e4 in mid‐life.
3. **Future directions**: Whether transentorhinal and hippocampal tau mediates the relationship between APOE e4 gene‐dose and perceptual discrimination remains to be established. Future research may also explore the utility of perceptual discrimination tasks in clinical trials targeting the preclinical stages of Alzheimer's disease.

Here, we tested if APOE e4 carriers show differences in simple and complex perceptual discrimination in mid‐life, using a remote online version of the “Greebles” task.^28^ Effects of APOE e4 gene dose are explored, facilitated by targeted recruitment of elevated numbers of APOE44 individuals compared to population prevalence.^29^ The lifetime risk of dementia is substantially higher in APOE44, relative to APOE34, with 60% of homozygous individuals receiving a diagnosis of AD by 85 years.^30^ Furthermore, a recent study reports AD neuropathology (including tau) increases dramatically in APOE44 individuals from age 50, with dementia symptoms emerging earlier (mean age 65.6 years) in this high‐risk group.^31^ As such, we predict a stepwise disadvantage in perceptual discrimination with the number of APOE e4 alleles carried. Such a difference would be consistent with enhanced early vulnerability to tau in this group.

## 2 METHODS

Hypotheses, experimental design, and statistical procedures were pre‐registered on the Open Science Framework in advance (https://doi.org/10.17605/OSF.IO/F67XJ).

### 2.1 Participants

Three‐hundred and thirteen middle‐aged adults (45–65 years old) were recruited from the NIHR BioResource volunteer database to take part in a large online study: The APOE Memory Bank. Exclusion criteria included any self‐reported dementia, neurological, or current psychiatric diagnoses. In addition, APOE e2 carriers were excluded due to the reported protective effects of this less common APOE variant against Alzheimer's disease.^32^, ^33^

Participants were selected with a bias towards APOE e4 carriers, with 5‐year age‐band and gender matched by APOE status (Table 1). Most participants (97.73%) self‐reported as white or white British. Upon entry to the study, all participants completed the Cognitive Change Index (CCI),^34^ as a marker of self‐perceived functional decline (Table 1). Part A of the CCI asks participants if they've noticed a decline in their cognition and whether this is cause for worry. Part B provides a score from 20 to 100 with higher values representing greater frequency of cognitive complaints.

**TABLE 1:** Demographic characteristics of participants grouped by APOE e4 carrier status and gene dose, including statistical tests of genotype group differences where relevant

	APOE e4 status				APOE e4 gene dose			
Parameter	APOE33	APOE e4+	χ ^2^	*p*	APOE34	APOE44	χ ^2^	*p‐*value
*n*	142	171			135	36		
Gender (%F)	52.81	50.29	0.11	0.740	51.11	47.22	0.37	0.831
Age‐band (years, *n*)			3.29	0.350			12.56	0.051
45–49	32	25			24	1		
50–54	30	41			36	5		
55–59	34	45			32	13		
60–65	45	60			43	17		
Immediate family history of dementia (%)	18.31	25.15	4.68	0.196	25.93	22.22	6.12	0.410
Degree‐level education (%)	53.52	55.56	0.09	0.770	54.81	58.33	0.28	0.869
CCI – part A								
Worried about decline (%)	11.97	15.79	2.60	0.272	18.15	5.56	9.23	0.055
CCI part B	27.6 ± 7.9	28.2 ± 9.3		0.758	28.5 ± 9.9	27.3 ± 6.4	0.10	0.952
Mean trial *n* classed as reaction time outlier	0.78	0.83	2.08	0.557	0.86	0.69	4.00	0.676

*Note*: Degree‐level education (%) refers to the percentage of participants reporting having at least a university undergraduate degree (higher education). Immediate family history (%) refers to the percentage of participants reporting having a parent or sibling with a dementia diagnosis. CCI part A refers to the percentage of participants saying they've noticed a decline in their cognition and are worried about it; part B presents mean score ± standard deviation (range of possible values = 20–100).

Abbreviation: APO, apolipoprotein E; CCI, Cognitive Change Index.

All participants gave informed electronic consent, including confirmation that they did not meet exclusion criteria for the study and acknowledgement that participants would not receive feedback on their APOE status. The study was approved by the University of Sussex Science & Technology Research Ethics Committee (ER/CLL6/2) and was completed in accordance with the ethical standards outlined in the 1964 Declaration of Helsinki (and its later amendments).

### 2.2 “Odd‐one‐out” task

**FIGURE 1:**
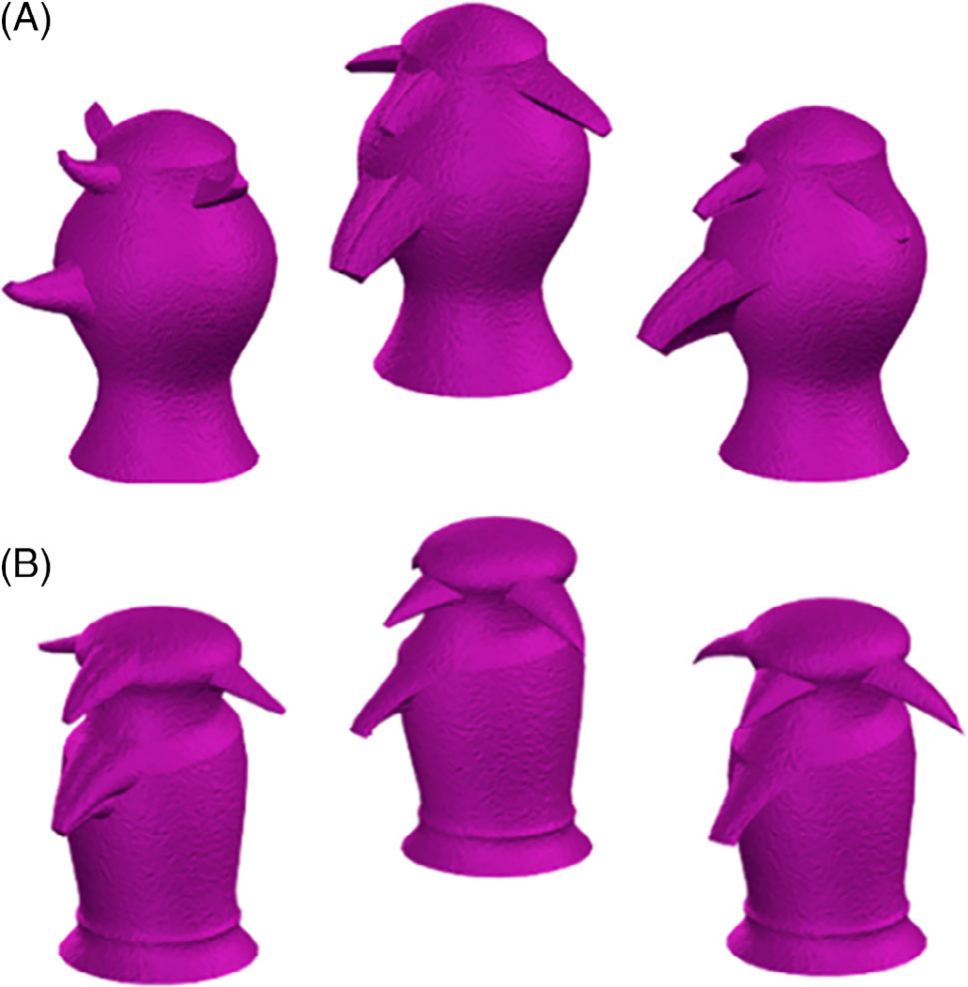
Example stimuli arrays in the Greebles “odd‐one‐out” task. Greebles in the low ambiguity condition (A) share a small proportion of perceptual features, relative to Greebles in the high ambiguity condition (B).

The “odd‐one‐out” task is a forced‐choice measure of perceptual discrimination. In each trial, participants were shown three computer‐generated, fictional objects (Greebles – http://www.tarrlab.org) from marginally different viewpoints (see Figure 1). Two of these objects are the same and one is different. Participants were asked to select the “odd‐one‐out,” indicating their choice by clicking on a button on the screen. There is no limit on how long participants can take to respond, however, participants were instructed to respond as accurately and as quickly as possible.

In the low ambiguity condition (24 trials), the presented Greebles^28^ share few perceptual features (e.g., the position of an appendage) and hence discriminating between them places relatively low demand on perceptual processes. In the high ambiguity condition (36 trials), exemplar Greebles are matched on several features (e.g., body shape, basic shape, and position of appendages). As such, discrimination requires a conjunction of features to be considered, exerting substantially greater demand on perceptual processes.

Participants were presented with 10 practice trials before undertaking the task, in which the correct answer and distinguishing features were highlighted to the participant after they had made their response. Test trials (*n *= 60) were presented in a random order. All participants completed the task in a remote, “home” environment via a subdomain on the lab website (d01.eventmemory.org). Access to the task was restricted to desktop or laptop computers.

### 2.3 Card‐sort task

Participants were asked to complete a card‐sort task as a baseline measure of speeded decision‐making. Task accuracy is presented here to help isolate genotype differences in complex perceptual discrimination from genotype differences in processing speed, attention, and executive function. Specifically, participants were asked to respond to a succession of playing card stimuli (two decks of 52 cards), displayed in a pseudorandom order on screen. In each trial, a card back is displayed for a 1000 ms, followed by a card face for 1000 ms. Participants were required to sort cards according to suit, pressing “ < ” for a heart and “ > ” for a spade, as quickly and accurately as possible. Participants are asked to give no response if presented with a diamond or a club.

### 2.4 Statistical analysis

#### 2.4.1 “Odd‐one‐out” task

Initially, data were screened for participants showing deviant task performance. No individuals met pre‐registered criteria for exclusion, namely, being classed as an outlier (defined as more than 3 standard deviations [SDs] from the population norm) in both accuracy (proportion) and mean response time (RT) in the low ambiguity condition. Five individuals (two APOE33, two APOE34, and one APOE44) were classed as outliers in terms of accuracy. However, re‐running analyses with these individuals removed did not qualitatively change outcomes from the study. Chi‐squared tests were used to test group‐differences in the distributions of gender, education, family history of dementia, and scores on the Cognitive Change Index – part A. Non‐parametric statistics (Wilcoxon Rank Sum, Kruskal‐Wallis) were used to screen group‐differences in the Cognitive Change Index (part B).

Frequentist generalized‐linear mixed effects models (GLMMs) were computed by maximum‐pseudolikelihood estimation in R (Package: lme4, version1 ‐1.32). Equivalent Bayesian models were estimated using the “BRMS” package in RStan (version 2.19.0). Frequentist statistics and Bayesian 95% credibility intervals (CIs) are reported for main effects and interaction terms, plus pairwise comparisons of interest (*p*‐values adjusted using Holm‐Bonferroni method for multiple comparisons). Bayesian CIs were computed by multiplying a contrast vector or matrix by all posterior samples returned by RStan. For contrasts with 1 degree of freedom, CIs were taken as the upper and lower limits that included the central 95% of contrasted samples. To produce a single interval for contrasts with more than 1 degree of freedom, contrasted posterior samples were multiplied by a rank‐1 unit vector coincident with the contracted posterior mean before the upper and lower limits were calculated.

Bayesian 95% CIs for model estimates enable greater consideration of the range of plausible effects, including the size, valence and precision of parameter estimates. Furthermore, as Frequentist and Bayesian analyses use different model‐fitting techniques, adopting these two complementary approaches confirms the robustness of statistical conclusions. In acknowledgement of open‐science best practices, model parameter estimates and standard errors for all analyses are uploaded to the project repository on the Open Science Framework, alongside anonymised trial‐level datasets and scripts for running GLMMs in R (https://osf.io/qns2r/).

##### Accuracy

The number of correct perceptual discriminations (k) for each participant, relative to the number of incorrect trials (trial n‐k) was modelled in two GLLMs as a binomial process with a logit link function such that parameter estimates encode the probability of a correct response on each trial. For Bayesian model estimates, the prior for the intercept term was set to a logistic distribution with μ=0 and s=1. Priors for all other fixed effects were set to a Cauchy distribution with a location parameter ($x_{0}$) of 0, and a scale parameter (γ) of $\sqrt{0.5}$ *. Model 1a, testing for an effect of e4 status, included the following fixed effects: (1) APOE e4 status (APOE33 = 0, APOE e4+ = 1); (2) Ambiguity (low = 0, high = 1); (3) z‐standardized 5‐year age‐band,—plus all possible interaction terms. Model 2a, testing for an effect of e4 gene dose, included the following fixed effects: (1) APOE e4 gene dose modelled as a factor with two dummy coded predators (APOE34 vs. APOE33, and APOE44 vs. APOE33); (2) Ambiguity (Low = 0, High = 1); (3) z‐standardized 5‐year age‐band,—plus all possible interaction terms. Note, APOE genotype haplotype was modelled as a non‐linear factor given that this structure yielded a lower Akaike Information Criterion (AIC) score (1373.0) when compared to a model that treated gene dose as a linear predictor (1374.1). Both Models 1a and 2a included random intercepts for each participant to account for inter‐individual variability in performance.

In addition to trial‐level GLMMs, a standardized difference score (z (% correct high ambiguity trials—% correct low ambiguity trials)) was extracted to provide a single metric of conjunction‐based perceptual discrimination after controlling for differences in basic perceptual ability, consistent with past literature (Gellersen et al., 2022). Genotype group differences were considered using a between‐groups *t*‐test (APOE33 vs. APOE e4+) and a one‐way analysis of variance (ANOVA; APOE33 vs. APOE34 vs. APOE44) with age included as a covariate. Complementary Bayesian models were computed to test the effect of APOE e4 status and APOE e4 gene dose. The prior for the intercept term was set to a normal distribution with μ=0 and s=1. Priors for all other fixed effects were set to a Cauchy distribution with a location parameter ($x_{0}$) of 0, and a scale parameter (γ) of 0.5.

##### RT

Prior to analysis, RTs more than 3 SDs away from the individual's personal mean RT were removed. This resulted in 251 RTs being removed from the dataset from across 194 individuals (84 APOE33, 90 APOE34, 20 APOE44; see Table 1). The proportion of removed trials per task did not significantly differ by APOE4 status (χ ^2^(3) = 2.08, *p *= 0.557), or APOE4 gene dose (χ ^2^(6) = 4.00, *p *= 0.676). Corresponding accuracy data were not removed for trials with outlier RTs. Trial RT (in seconds) for correct decisions was subsequently modelled in two GLMMs with a log link function and the gamma distribution. For Bayesian models, the prior for the intercept term was set to a lognormal distribution with μ=0 and σ=1. Priors for all other fixed effects were set to a Cauchy distribution with a location parameter ($x_{0}$) of 0, and a scale parameter (γ) of $\sqrt{0.5}$. Model 1b included the same fixed‐ and random‐effects predictors as Model 1a (for accuracy, see above). Similarly, model 2b had the same fixed‐ and random‐effects predictors as Model 2a with the exception that APOE haplotype was modelled by a single linear predictor indicating gene dose (APOE33 = 0, APOE34 = 1, APOE44 = 2). This was done as a single linear predictor yielded a better fit to the data (AIC = 79124.6) compared with a model that treated APOE haplotype as a categorical variable (AIC = 79129.2).

##### Speed‐accuracy trade‐off

Exploratory analyses (not pre‐registered) extracted the correlation between mean RT (correct responses) and mean proportion accuracy in the high ambiguity condition for each genotype group. This additional analysis was completed to examine if group differences in strategy for task completion (e.g., being slower to maintain accuracy) accounted for Genotype or Age effects.

#### 2.4.2 Card‐sort task

Trial‐level sort accuracy was modelled in two GLLMs as a binomial process with a logit link function such that parameter estimates encode the probability of a correct response on each trial. The first model included APOE e4 status (APOE33 = 0, APOE e4+ = 1), z‐standardized 5‐year age‐band, and the interaction term as fixed predictors, plus random intercepts for each participant. The second model was identical, except replacing APOE e4 status with APOE e4 gene dose (linear predictor) as a fixed effect.

## 3 RESULTS

The distribution of ages is equivalent between APOE33 and APOE e4+ individuals (χ ^2^(1) = 3.29, *p *= 0.350); however, there is a trend‐level difference in the distribution of age‐groups by APOE e4 gene dose (χ ^2^(2) = 12.56, *p *= 0.051, with a higher proportion of the APOE44 group being in the 60–65 years age bracket. Genotype groups were also equivalent in gender, the proportion of participants reporting university degree‐level education, and the proportion of participants reporting an immediate (parent or sibling) family history of dementia (*p *> 0.05). Including gender in reported statistical models did not influence reported genotype effects (see Supplementary Materials for effects of Gender on task performance, including Figure S1 and S2).

There was no significant difference in the distribution of responses to the CCI – part A by APOE e4 status (χ ^2^(1) = 2.60, *p *= 0.272) or gene dose (χ ^2^(2) = 9.23, *p *= 0.055). In addition, there was no difference by APOE e4 status (*W = *11896*, p = 0*.758) or gene dose (χ ^2^(2) = 0.10, *p *= 0.952) on scores on the CCI – part B. Demographic characteristics for the sample are shown in Table 1. Descriptive statistics for performance on the Greebles “odd‐one‐out task” and card‐sort task are shown in Table 2.

**TABLE 2:** Descriptive statistics of performance accuracy (%) and mean RT (correct trials only) on the Greebles “odd‐one‐out” task and card‐sort decision‐making task, shown by APOE e4 status and APOE e4 gene dose

	E4 status		E4 gene dose	
Parameter	APOE33	APOE e4+	APOE34	APOE44
**Greebles**				
Accuracy (%)				
Low ambiguity	98.83 ± 2.87	99.20 ± 2.23	99.28 ± 1.94	98.50 ± 3.01
High ambiguity	95.74 ± 6.67	95.52 ± 6.80	96.28 ± 5.70	92.97 ± 9.46
RT (s)				
Low ambiguity	4.75 ± 2.61	5.17 ± 3.99	5.24 ± 4.23	4.92 ± 2.90
High ambiguity	6.46 ± 4.33	7.12 ± 5.63	7.18 ± 5.88	6.90 ± 4.54
**Card‐sort**				
Accuracy (%)	96.18 ± 2.25	96.41 ± 1.95	96.29 ± 1.98	96.90 ± 2.25

Abbreviations: APO, apolipoprotein E; RT, response time.

### 3.1 Effect of e4 status on response accuracy

Model 1a (626 observations of k – successful discriminations) reports a significant main effect of increasing trial ambiguity on accuracy, *F*(1, 617) = 140.90, *p *< 0.001; Bayesian 95% CI: [‐3.77, ‐2.72]. As expected, the probability of a correct response is higher for low relative to high ambiguity trials. In addition, there is a significant main effect of Age, *F*(1, 617) = 10.86, *p *= 0.001; Bayesian 95% CI: [‐1.89, ‐0.28] (see Table S1 for descriptive statistics of task accuracy by 5‐year age‐band). The main effect of e4 status, *F*(1, 617) = 0.03, *p = 0*.865; Bayesian 95% CI: [‐0.49, 0.98], however, was non‐significant, as were all interaction terms in the model (*p *> 0.05; Bayesian 95% CIs cross 0). When considering *z‐*standardized difference scores, there was no significant difference between APOE33 (*M = 0*.058 ± 0.964) and APOE e4+ (*M = *‐0.048 ± 1.030); *F*(1, 305) = 0.87, *p *= 0.352, *η^2^_ρ_* = 0.00, Bayesian 95% CI: [‐0.30, 0.14].

### 3.2 Effect of e4 gene dose on response accuracy

Model 2a (626 observations of k – successful discriminations) again reports significant main effects of trial Ambiguity, *F*(1, 613) = 138.03, *p* < 0.001, Bayesian 95% CU: [‐5.84, ‐4.05] and Age, *F*(1, 613) = 11.11, *p *< 0.001, Bayesian 95% CI: [‐4.56, ‐0.67]. The main effect of APOE gene dose is also significant, *F*(2,613) = 3.75, *p *= 0.025, Bayesian 95% CI: [0.07, 1.90]. Planned contrasts (holm‐Bonferroni adjusted; Bayesian) report a significant difference between APOE34 and APOE44 individuals (*p *= 0.010, critical α = 0.016, Bayesian 95% CI: [‐2.62, ‐0.15]).

**FIGURE 2:**
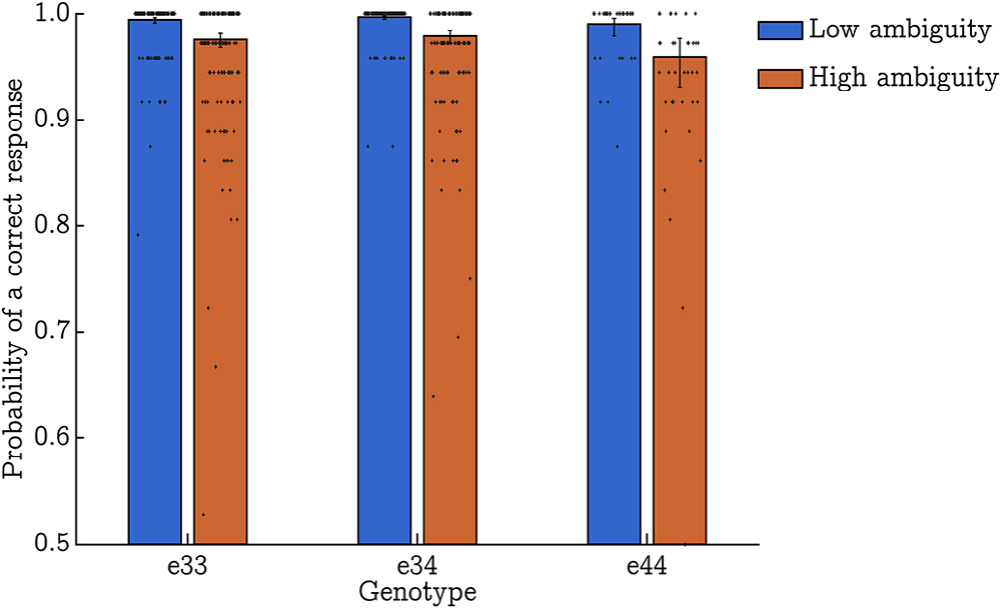
Frequentist model estimates of probability of correct response on low and high ambiguity trials on the Greebles “odd‐one‐out” task, separated by apolipoprotein E e4 gene dose. Error bars represent 95% confidence intervals of model estimates. Overlaid scatterpoints represent estimates for each individual (number of successes [k]/trials [n]).

Results are not indicative of a significant pairwise difference between APOE33 and APOE34 (*p *= 0.096, critical α = 0.013, Bayesian 95% CI: [‐0.15, 0.1.44] or between APOE33 and APOE44 (*p *= 0.116, critical α = 0.010, Bayesian 95% CI: [‐1.91, 0.37]). The Ambiguity x APOE gene dose interaction (Figure 2) was non‐significant (*p *= 0.258, Bayesian 95% CI: [‐0.18, 1.02], as were all other interaction terms in the model (*p *> 0.05, Bayesian 95% CI include 0). The Ambiguity x Age x APOE gene dose interaction (*p *= 0.199, Bayesian 95% CI: [‐0.26, 1.20]) is shown in Figure S3.

GLMM 2a reports a significant main effect of gene dose on task accuracy (*p *= 0.025, Bayesian 95% CI: [0.07, 1.90]), despite ceiling effects in the low ambiguity condition. Critically, these ceiling effects also impact the model's ability to separate out variance attributable to interactions versus main effects. However, whereas the Dose x Ambiguity interaction was non‐significant in GLMM 2a (*p *= 0.258, Bayesian 95% CI: [‐0.18, 1.02]), there was a significant effect of APOE e4 gene dose on Z‐standardized difference scores which reflect the cost of increasing trial ambiguity on task accuracy, *F*(2, 301) = 3.88, *p *= 0.022, *η^2^_ρ_* = 0.03. Following Holm‐Bonferroni adjustment, post‐hoc comparisons suggested there was a significant difference in Z‐standardized difference scores between e33 carriers (*M = 0*.058 ± 0.964) and e44 carriers (*M = *‐0.433 ± 1.520; *p *= 0.025, Bayesian 95% CI: [‐0.83, ‐0.02]) and e44 carriers and e34 carriers (*M = 0*.055 ± 0.830; *p *= 0.025, Bayesian 95% CI: [‐0.80, ‐0.06]). The main effect of Age and Age x APOE haplotype interaction were both non‐significant (*p *> 0.05) on z‐standardized difference scores. Note, analyses of Z‐standardized difference scores were pre‐registered in advance and replicate the methods of a past paper utilizing this paradigm.^9^

### 3.3 Effect of e4 status on RT

**FIGURE 3:**
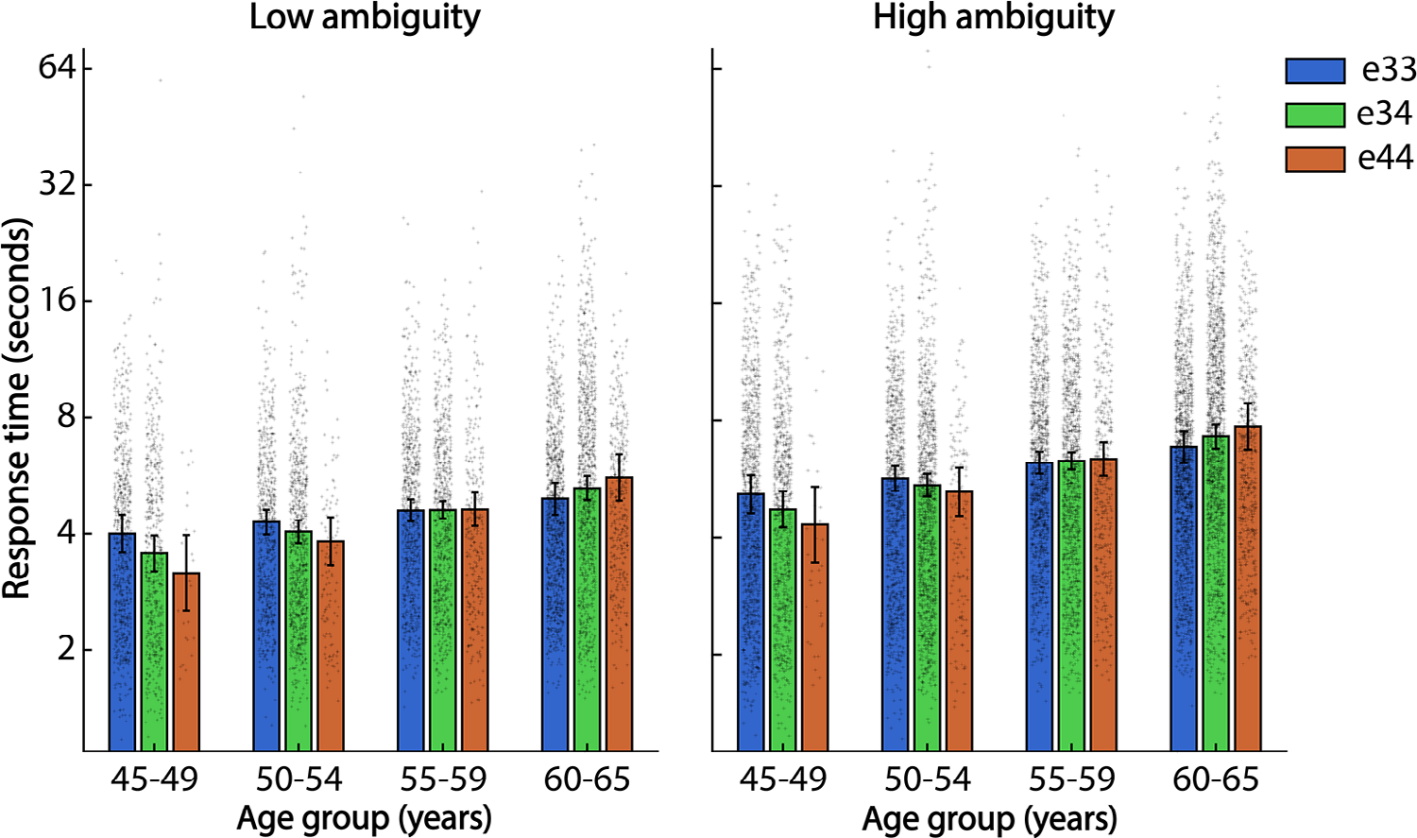
Frequentist model estimates of response time (RT) for correct perceptual judgments on low and high ambiguity trials, separated by apolipoprotein E e4 gene dose and 5‐year age‐band. Overlaid scatterpoints represents RT recordings from individual task trials.

Model 1b (17962 observations) reports a significant main effect of trial ambiguity on RT, *F*(1, 17952) = 1895.95, *p *< 0.001; Bayesian 95% CI: [0.58, 0.63]. As expected, RTs are slower in the high ambiguity relative to the low ambiguity condition (Table 2). There is also a significant main effect of Age, *F*(1, 17952) = 112.89, *p *< 0.001, Bayesian 95% CI: [0.36, 0.65], with RTs slowing with increasing age (see Table S1 for descriptive statistics of RT by 5‐year age‐band). However, the main effect of APOE e4 status is non‐significant, *F*(1, 17952) = 1.53, *p *= 2.16, Bayesian 95% CI: [‐0.10, 0.22]. There was a significant Age x APOE e4 status interaction, *F*(1,17952) = 12.95, *p *< 0.001; Bayesian 95% CI: [0.01, 0.33]. As can be seen in Figure 3, in the younger age‐group, the APOE e4+ responded faster than the APOE e33 control group across both low and high ambiguity trials, whereas this pattern was reversed in adults aged 60 years and older. The Ambiguity x Age interaction was also significant, *F*(1,17952) = 8.79, *p *= 0.003; Bayesian 95% CI: [0.02, 0.07], with age‐related slowing greater on high ambiguity relative to low ambiguity trials. Both the Ambiguity x APOE e4 status and 3‐way Ambiguity x Age x APOE e4 status interactions, however, were non‐significant (*p *> 0.05, Bayesian 95% CI include 0].

### 3.4 Effect of e4 gene dose on RT

As in Model 1b, Model 2b (17962 observations) reports a significant main effects of trial Ambiguity on RT, *F*(1, 17952) = 1895.46, *p *< 0.001; Bayesian 95% CI: [0.58, 0.63] and Age, *F*(1, 17952) = 112.32, *p *< 0.001; Bayesian 95% CI: [0.32, 0.64]. The main effect of Gene Dose, however, is non‐significant, *F*(1, 17952) = 0.09, *p *= 0.758; Bayesian 95% CI: [‐0.13, 11]. There was a significant Age by Ambiguity interaction, *F*(1, 17952) = 8.79, *p *= 0.003; Bayesian 95% CI: [0.01, 0.07] and Age x APO*E* e4 gene dose interaction, *F*(1,17952) = 9.75, *p *= 0.002; Bayesian 95% CI: [0.00, 0.25] (Figure 3). Genotype effects on RT reversed with increasing age, and this effect was stronger in APOE e4 homozygous group relative to heterozygous individuals (see Figure 3). All other interaction terms were non‐significant (*p *> 0.05; Bayesian CIs cross 0).

### 3.5 Speed‐accuracy trade‐off

There was a small, non‐significant correlation between proportion correct (accuracy) and mean RT on correct trials in all three genotype groups: APOE33 ‐ ρ = ‐0.16, *p = 0*.052, 95% CI: [‐0.32, 0.00]; APOE34 ‐ ρ = ‐0.08, *p = 0*.354, 95% CI: [‐0.24, 0.09]; APOE44 ‐ ρ = 0.04, *p = 0*.827, 95% CI: [‐0.30, 0.36]. Further, these correlations do not significantly differ from each other (z < 1.05, *p* > 0.14, for all comparisons). This suggests there were no clear differential speed/accuracy strategies adopted between genotypes.

### 3.6 Card‐sort performance

Main effects of APOE e4 status, Age (z‐standardized), plus the APOE e4 status x Age interaction on card‐sort accuracy were all non‐significant (*p *> 0.05). When including APOE4 gene dose as a fixed predictor in the model, main effects and interactions terms were also non‐significant (*p >* *0*.05). See Table 2 for descriptive statistics of task performance by genotype group.

## 4 DISCUSSION

The present study aimed to establish whether possession of an APOE e4 genetic risk variant for dementia is associated with impaired perceptual discrimination in mid‐life. In addition, the number of APOE44 individuals present in this study facilitated novel exploration of gene‐dose effects. Methods employed a remote Greebles “odd‐one‐out” task, with performance on this task correlated with structure and function of the MTL^11^—a brain‐region compromised in early AD.

Current results show only APOE44 individuals are impaired in their perceptual discrimination abilities by mid‐life, with this highest‐risk group being less accurate in their judgments than APOE34 individuals and an APOE33 control group. Furthermore, although our interpretation of the Ambiguity by Genotype interaction term is limited by ceiling effects, standardized difference scores between accuracy on high and low ambiguity trials indicate APOE44 individuals show greater cost of increasing perceptual overlap. As APOE44 individuals showed comparable accuracy on an attentionally‐demanding card‐sort task, characterized by lower perceptual demands, deficits appear specific to tasks which challenge feature‐based object discrimination. Cross‐sectional analyses revealed slopes of age‐related slowing of reaction times increased linearly with the number of APOE e4 alleles carried, across both low and high ambiguity trials. This may indicate that, although APOE34 carriers show intact cognitive performance into the sixth decade, reduced efficiency of perceptual judgments was recorded in the oldest age‐band (60–65 years) and may reflect subtle disadvantages emerging with increasing age.

Observed performance disadvantages in mid‐age APOE44 are consistent with prior studies reporting perceptual discrimination tasks are sensitive to heightened familial risk of AD in mid‐life,^26^ plus distinguish older adults at risk of converting to dementia based on neuropsychological test scores.^9^, ^10^ This supports the utility of paradigms which tax high‐level perceptual processing as early cognitive markers of AD risk. Current results are not inconsistent with past research evidencing equivalent perceptual discrimination in young heterozygous APOE e4 carriers^25^ as accuracy was maintained in our APOE34 group, coupled with quicker RTs until age 60. This previous neuroimaging study,^25^ however, did not include APOE44 individuals, nor examine speed of perceptual decision‐making as an outcome of interest. Given the pattern of age‐related change in perceptual processing speed observed here, younger APOE34 and APOE44 may show stepwise advantages in high‐speed decision‐making in early adulthood.

APOE34 carriers in the current study showed intact accuracy of perceptual judgments. Reported cognitive effects of APOE e4 vary across the lifespan and by cognitive domain,^4^, ^20^, ^35^ with some studies finding APOE e4 advantages in MTL‐dependent^36^, ^37^ and speed‐dependent tasks^38^, ^39^ in younger years. Indeed, one study reports APOE4 advantages on an object‐location precision memory task at age 70 in a sample of predominantly heterozygous e4 carriers, particularly in the context of higher amyloid.^40^ Authors interpreted this pattern of performance to reflect increased utilization of compensatory executive resources in APOE e4+ as neurological systems are put under increased stress. Future research may observe disadvantages on the Greebles “odd‐one‐out” task emerging in APOE34 individuals older than 65 years or utilize longitudinal data to capture possible genotype differences in change over time. This may occur particularly in the context of tasks placing increased demands on attentional processing, with evidence of accelerated age‐relating slowing of RTs present in this study.

This study provides the first evidence that APOE e4 deficits in perceptual discrimination emerge as a function of the number of risk alleles carried in mid‐life, with disadvantages primarily seen for homozygote individuals. Due to the relatively low (∼ 2%) prevalence of APOE44 individuals in the population,^41^ few lab‐based studies have considered the impact of APOE e4 zygosity. Paradigms which place significant challenge on MTL‐function, however, support the presence of gene‐dose effects from the fifth decade. Specifically, a linear disadvantageous effect of APOE e4 was observed in cognitively healthy adults aged 40–60 years old when recall performance was stressed by a 7‐day delay between learning and test (as opposed to 30‐min).^42^ In the same sample, a detrimental effect of APOE e4 zygosity was also reported in the ability to harness audio memory to guide attention.^43^ Standardized neuropsychological assessment is more commonly employed by large‐scale birth cohorts. Examination of longitudinal trends suggest only APOE44 individuals show a significant decline in episodic memory from age 43–69 years (relative to APOE33 counterparts),^44^ although a comparable, non‐significant trend was seen when looking at APOE34 carriers. This effect was not seen on tests of processing speed, suggesting disadvantageous effects of APOE4 gene dose may be heightened in tasks probing tau‐sensitive MTL‐regions.

The PRc is one brain region involved in our ability to process objects as a conjunction of perceptual features,^8^, ^45^ for example the high‐ambiguity trials in this Greebles “odd‐one‐out” task. APOE44 individuals showed increased cost of higher trial ambiguity, which may suggest altered PRc function. Given that the PRc is the site of earliest neurofibrillary tau deposition, reported genotype differences may indicate APOE44 individuals show premature, toxic‐gain of tau neuropathology.^22^, ^23^, ^24^ Recent evidence collating *post mortem* neuropathology, clinical, and biomarker data from five cohorts suggests the predictability of biomarker trajectories and symptom onset, plus near‐complete penetrance of biomarker positivity in homozygous individuals is comparable to autosomal forms of AD.^30^ Indeed, models of biomarker trajectories suggest that tau positivity begins to increase substantially in APOE44 individuals around age 55, in agreement with theoretical accounts of the current results. As such, perceptual discrimination errors may reflect that, even by mid‐adulthood, APOE44 represent an AD prodrome. Perceptual disadvantages in APOE44 carriers may also reflect early functional reorganization of MTL network, with increasing age correlating with poorer mnemonic discrimination at short memory delays and reduced specialization of PRc and alERc for processing objects relative to scenes.^13^

APOE44 carriers, and to a lesser extent APOE34 carriers showed increased age‐related slowing in perceptual judgments. Curiously, APOE e4 carriers as a collective appeared faster in early mid‐life. This may confer support for the antagonistic pleiotropy account of APOE; that carriers show advantages in tasks requiring sustained attention in younger years.^38^, ^39^, ^46^ This result was consistent across low and high‐ambiguity trials, suggesting efficiency may reflect a domain‐general process like processing speed, rather than representing the integrity of the PRc. In older adults, processing speed was reported to decline as a function of APOE e4 zygosity,^47^ however, gene‐dose effects in this domain were not reported in mid‐age.^44^ There has been limited consideration of reaction time in perceptual judgment tasks; however, Jiang and colleagues^10^ reported no difference in discrimination times between cognitively healthy older adults and those with impairments in global cognition. In the current study there was no suggestion that genotype groups were preferentially prioritizing accuracy at the expense of RT or vice versa. However, as the Greebles perceptual discrimination task is sensitive to age‐effects by mid‐life [48], it may be that APOE e4 carriers are collectively more vulnerable to accelerated ageing.

Accuracy in this study was close to ceiling for both low and high ambiguity trials which may have limited our sensitivity to genotype effects, particularly in the context of the interaction with increasing trial ambiguity. Calculation of differences scores add to our understanding of the cost of increasing perceptual feature overlap. Stimuli are central to perceptual discrimination tasks; “Greeble” objects are relatively humanoid in appearance which may benefit our ability to distinguish between them. A recent study manipulated the spatial arrangement and type of feature extracted from letter stimuli to create a more abstract, six‐item “odd‐one‐out” task.^8^ Accuracy on this task distinguished healthy older adults from individuals diagnosed with mild AD and correlated with volume of the PRc and entorhinal cortex. Furthermore, the extent to which viewpoint differs when distinguishing between stimuli is correlated with increased blood‐oxygen‐level‐dependent (BOLD) response in the PRc,^11^ suggesting mental rotation may contribute to differences in task performance.

Future research may consider whether the sensitivity of perceptual judgment tasks could be elevated by further challenging detailed perceptual processing and the role of mental rotation in task performance.

The current study did not screen for genotype differences in global cognition; hence, it may be that APOE44 individuals showing disadvantaged performance on the “odd‐one‐out” task show broader cognitive and functional difficulties relevant for dementia diagnosis. Descriptive statistics of “odd‐one‐out” performance in the current sample, however, are equivalent, if not better, than mid‐age and older‐aged adults established to be cognitively healthy.^9^, ^27^ Furthermore, there was also no evidence APOE44 carriers were differentially experiencing subjective cognitive impairment, a risk factor for future dementia diagnosis.^34^

It must be acknowledged that, although recruiting through NIHR BioResource allowed us access to a greater number of APOE44 individuals than prior experimental studies, the study remained underpowered to reliably detect small (Cohen's f = 0.15) effects of APOE e4 gene dose. In particular, the small number of APOE44 individuals present when stratifying by 5‐year age‐band limits interrogation of Dose by Age interactions. Although genotype groups were gender‐matched with a range of educational backgrounds, our sample was not ethnically diverse, and no information was collected on participant's ophthalmic health. Embedding paradigms hypothesized to be sensitive to “preclinical” neurodegenerative disease, such as MTL‐dependent perceptual discrimination tasks, in biomarker rich, longitudinal population cohorts will build understanding of how risk manifests across the lifespan. Digital, scalable, and remote assessments can accelerate this work. Furthermore, such studies can further our understanding of how cognitive assessments can supplement prediction of very early, sub‐threshold biomarker development.^2^

To conclude, perceptual discrimination tasks appear sensitive to the deleterious effects of APOE44 by mid‐life. This potentially reflects increased vulnerability of this group to premature tau neuropathology, with recent evidence reporting nearly all APOE44 were biomarker positive by age 55.^30^ Mid‐adulthood is a time of strategic importance for slowing the development of neurodegenerative conditions. Future research can harness measures of perceptual discrimination to facilitate cost‐effective screening for clinical trials targeting preclinical AD, plus promote individualized interventions to mitigate risk prevention. First, further evidence elucidating the relationship between perceptual discrimination, tau neuropathology, and APOE in mid‐life is needed.

## CONFLICT OF INTEREST STATEMENT

Declarations of interest: none (C.L., S.B., J.D., J.R., C.B.). Author disclosures are available in the supporting information.

## CONSENT STATEMENT

All participants provided informed consent prior to taking part in this research.

## Supporting information



Supporting Information

Supporting Information

## Footnotes

* Prior $\gamma =\sqrt{0.5}$ is in keeping with expectation of medium‐sized effects.
